# Glimpse on 21st century new phobias; a predictive model of nomophobia

**DOI:** 10.3389/fpubh.2023.1252099

**Published:** 2023-12-21

**Authors:** Ciprian Marius Ceobanu, Andrei Lucian Marian, Roxana Apostolache

**Affiliations:** Teacher Training Department, Faculty of Psychology and Educational Sciences, “Alexandru Ioan Cuza” University of Iasi, Iași, Romania

**Keywords:** techno phobias, nomophobia, rumination, fear of missing out, non-pathological compulsions, mindfulness

## Abstract

The main purpose of this study was to examine the explanatory power of a predictive model of nomophobia consisting of rumination, fear of missing out (FoMO), mindfulness and non-pathological compulsions. The research involved a cross-sectional design exploring the prevalence of nomophobia in a Romanian university students’ cohort. The quantitative methodology was used to collect and analyse the data obtained from all the respondents. Researchers adapted and pretested the questionnaire NMP-Q, before distributing it to 194 university students. SPSS (V. 20) and Hayes’s PROCESS tool were used to analyse the data. The findings demonstrated that the above-mentioned psychological variables have a direct and significant relationship with nomophobia. Specifically, within the multidimensional mechanism that explains nomophobia, fear of missing out (FoMO), non-pathological compulsions, and rumination, included in the predictive model in this order, played the most important role, as together cover 34% of nomophobia variance. Furthermore, the fear of missing out has the highest explanatory contribution to nomophobia. The current study gives a better understanding of the dynamics of nomophobia in young people by focusing on psychological factors that play an important role in this phenomenon.

## 1 Introduction

Twenty years ago, probably no one would have thought that people can become addicted to their mobile phones and feel a serious state of stress when the device does not perform the expected functions. It is largely accepted that they serve purposes other than only communication, since information and entertainment are helping users to fulfill demands such as learning, developing individual skills, safety, and the need for social relationships (1). Frequent use of technology encourages recurrent and systematic monitoring of social media and texts and the consequences of this trend of frequent smartphone use could include disruptions in the current activity and a rise in anxiety (2), may have long-term effects resulting in personality disorders and perhaps exacerbating pre-existing problems e.g., “obsessive-compulsive personality disorder, social interaction anxiety, internet or smartphone addiction” [(3), p. 157]. While technology has undoubtedly improved our lives in many ways, excessive dependence on smartphones leading to nomophobia can have detrimental effects on mental health and overall wellbeing.

All these, suggests that technology has become a new source of unconscious and implicit reactions (4) giving rise to new pathologies – *techno-pathologies* (5). The overuse of digital devices could be conceived as a specific type of addiction and there are experts that talk about *fear of missing out* (6), *fear of being offline* and *nomophobia* as “forms of anxiety that border on obsession or compulsion” [(7), p. 3]. Other research has demonstrated that smartphones may result in compulsive checking behaviors, compulsive usage (8) and that smartphones can lead to addiction (9); extensive use of the mobile phone may have adverse consequences, materialized in increased depression levels, high anxiety, and poor sleep quality (10).

Some new emerging mental health problems can be added to this picture. For instance, *textaphrenia or textiety* (11), *phantom phone signals-ringxiety* (5), *netlessphobia* (12, 13) allows us to expand the image of 21st century digital diseases, that all are related to the use of mobile phone.

It is known that excessive dependence on smartphones (which can lead to nomophobia) can have adverse effects on mental health. It might contribute to increased stress, anxiety, and even depression due to factors like constant connectivity, social comparison, fear of missing out (FOMO), and decreased real-world interactions. Furthermore, nomophobia can significantly impact overall wellbeing. Reliance on smartphones at the expense of real-world experiences, relationships, and self-care can lead to a diminished sense of satisfaction and fulfillment in life. Excessive screen time can also affect sleep quality, physical health, and emotional balance, thus affecting overall wellbeing.

Over the last years, several authors have been concerned with identifying the psychological factors that play a significant role in explaining nomophobia. In other words, they looked to decipher the dilemma of the co-occurrence of nomophobia with certain states or stable personality traits. In this respect, some direct association between different variables and nomophobia have been observed, but also it was evaluated the strength of the interaction terms in order to explain variance beyond that can be explained by the main effects in a regression equation. Thus, several studies have tried to map direct, associative relationships between psychological factors and nomophobia *discomfort, anxiety, nervousness* and *nomophobia* (14), *separation anxiety* and *nomophobia* (15), *higher levels of fear, discomfort*, *avoidant attachment* and *nomophobia* (16), *rumination* and *nomophobia* (17), *FoMO,* and *nomophobia* (18), *mindfulness* and nomophobia (19), while others have proposed multiple regression models to capture the explanatory complexity of the phenomenon. For instance, the regression model proposed by Argumosa-Villar et al. (20) includes *self-esteem, extraversion, conscientiousness and emotional stability* as factors that significantly predicts nomophobia. The previous studies indicate that the set of psychological variables associated with the usage of mobile phones can be conceived as a complex relational structure.

Based on Field’s idea (21) according to which multiple hierarchical regression is the best way to build explanatory models of the outcome variables, the current study proposes a predictive model of nomophobia that includes *rumination, fear of missing out (FoMO), mindfulness, and non-pathological compulsions*. More than that, being a multi-faceted phenomenon, nomophobia could also be explained by indirect effects. In this respect, this study aims to investigate the mediation effect of fear of missing out (FoMO) on the relationship between rumination and nomophobia.

## 2 Theoretical background

### 2.1 Nomophobia and risk factors for problematic use of smartphone

The term *nomophobia* was devised in the Anglo-Saxon cultural space and comes from the expression “no mobile phone phobia’’ (22), which refers to the fear of not being in possession of your mobile phone or losing mobile phone contact. Nomophobia was described through four dimensions [(23), p. 130]:*“not being able to communicate, losing connectedness, not being able to access information, giving up convenience.”*

The pain or distress brought on by not having a smartphone available is known as being a *situational phobia* linked to *agoraphobia* (24). According to some authors (22, 25) the impact of ICT has favored the formation of new behaviors in people’s daily lives. Although its incorporation has streamlined and improved daily activities and provided benefits for people (26), smartphones also have caused addiction-related issues and even the emergence of psychological illnesses (nomophobia included) (27, 28). When separated from their smartphone for a short period of time and being unable to answer an incoming call, the subjects of some research (29, 30) experienced nomophobia in tandem with negative affect that leads them to elevated heart rate, blood pressure, and anxiety.

Other authors (14) connect nomophobia with a certain level of discomfort and anxiety caused by the lack of a smartphone. Recent researches proved that nomophobia positively correlates with neuroticism, attachment anxiety and loneliness (31). There are researchers (32) that place the *nomophobia* among the separation anxieties whereby mobile phones users experience a feeling of loss when their device is absent. Han and colleagues [(15), p. 2] suggest that separation anxiety (as described in literature) is linked to the “proximity-seeking tendency” toward the mobile device and is originated in extending one’s identity onto smartphones through the use of different ways of communication and social interaction.

As to the risk factors associated with nomophobia, different studies emphasize two main categories: socio-demographic and psychological factors. Most of the studies related to nomophobia focuses on university students (25, 33–40) and indicates that adolescents and young adults are more likely to be exposed to the negative effects of nomophobia. Emotionally dependent individuals and those that seeks for extra attention in the relationship, present higher levels of discomfort or fear. It was documented that “gender has a differential impact on the relationship between avoidant attachment and nomophobia” [(16), p. 1]. Nomophobia is predicted by several psychological factors such as: “self-esteem, extraversion, conscientiousness and emotional stability” [(20), p. 132].

Related to socio-demographic factors, in particular gender differences in the occurrence of nomophobia, some authors [(41), p. 19] highlighted that “females and young people seem to be more vulnerable to nomophobia.” In the same sense, Arpaci (42) reported statistically significant differences in nomophobia between women and men, wherein the female subjects outperformed the male subjects. Complementary, a study involving undergraduate students from Pakistan (40), explained that gender differences in NMP-Q questionnaire scores, were clearly influenced by the women subjects that demonstrates great levels of nomophobia.

The results indicate that the majority of studies investigating the incidence of nomophobia, primarily in teenagers and college students, are descriptive, nonexperimental, and cross-sectional in nature. These studies indicate that the current research is at an exploratory phase. An overall look upon the current researches suggests that nomophobia induces anxiety and stress and has a negative effect on personality, self-esteem and academic performance, leading to physical and mental health problems. All in all, this might be considered a health issue that has an adverse influence on the individual, leading to behavioral, physical, and psychological issues.

### 2.2 Rumination and nomophobia

The main psychological determinants of nomophobia namely anxiety, stress, fear, discomfort – are the triggers used by application developers and smartphones manufactures to exploit the user’s social and psychological weaknesses (43). The rapid distraction from current activities (mental disengagement) that mobile phones offer becomes a positive reinforcement that encourages the need to use smartphone because the human mind is predisposed to ruminating (44). Rumination – the tendency of an individual to think passively and repetitively about personal current feeling states, own characteristics and causes of these feelings (45) is considered to be a method of coping with negative moods that implies focusing on negative emotions (46). As a propensity to perseverate on the causes, experiences and implications of dysphoric emotions – rumination has received attention as a maladaptive emotion regulation response that is linked to prolonged dysphoric mood and depression risk (47, 48) and it was associated with anxiety (49) and with the distortion of emotional clarity (50). Because of the multiple negative consequences, it can induce (51), rumination is considered to be a major risk factor for depression and anxiety symptoms in both adolescents and adults (52, 53).

Rumination extensively predicted excessive smartphone use (44) but there is a scarcity of research examining the mechanisms and correlations that underly this relation (44). It seems that a general way of thinking based on exacerbating repetitive and intrusive negative thoughts could easily facilitate the path to nomophobia. Also, factors that could be put in relation with rumination mechanisms (e.g., “loneliness, social avoidance, and eccentricity”) significantly predicted nomophobia [(17), p. 1]. Rumination was found as a mediator for mobile phone addiction (54) and a predictor of smartphone addiction before going to sleep (55). Other individual characteristics such as emotional adaptability have an important effect on nomophobia (36).

### 2.3 FoMO (fear of missing out) and nomophobia

Being closely linked to the presence of the social networks, FoMO (fear of missing out) defines a state of uncertainty related to the fear of missing out on social network updates. The dual nature of the social media (on the one hand there are lots of opportunities for interaction, on the other hand these opportunities can be overwhelming in terms of time needed to pursue all the broadcast information) leads to this new type of online behavior.

FoMO is related to the desire to stay continuously online to witness to what others are doing and it was “defined as a pervasive apprehension” [(56), p. 1841] that other cyber-pals might have in terms of rewarding experiences while the subject is absent. The same authors (56) underlined that individual have low overall mood, low levels of welfare and life satisfaction. A recent study (57) has concluded that FoMO is directly related to depression, anxiety, and boredom, and a qualitative analysis on FoMO (58) reveals that the subject’s main concern is to avoid failure to be up to date with the latest news. After the massive entrance of the smartphones into our lives, the negative consequences of FoMO bring further proof that supports the existence of this type of addiction which leads people to spending a lot of time on social networks out of a fear of missing out.

The landscape of smartphone addiction is very extended and as a result, we cannot identify a definitive definition or a specific conceptualization of smartphone addiction. In this respect, there are papers that try to answer to nosology questions regarding smartphone addiction and, in this respect, the orientation is to use the non-pathological approach (59). Obviously, this is a matter of duration in using smartphones and terms such as *excessive use* or *overuse* of the smartphone, as well as *problematic* smartphone use have been proposed to replace the term of, “smartphone addiction” (60).

Other research findings (18) indicate that the students’ degrees of nomophobia and FoMO are above average. The above-mentioned authors show that there is “a moderately significant association and a positive direct relationship between fear of missing out and nomophobia, and that fear of missing out accounts for 30% of nomophobia” [(18), p. 16]. The results also reveal statistically significant differences in the levels of nomophobia and FoMO across students as well as variances in their demographic characteristics.

### 2.4 Mindfulness as positive factor to reduce nomophobia

People with high trait mindfulness tend to act consciously and attentively in their daily activities and they are generally non-reactive, non-judgmental, and conscious of their thoughts and emotions (61). In relation with *nomophobia*, mindfulness can act like a factor that can reduce tendencies for addictive and anxious behaviors (62).

Mindfulness is generally associated with positive emotional outcomes (62); as a consequence, a significant number of studies were produced along this line (63). There is research that suggests that while mindfulness increases, the effect of nomophobia on addictive smartphone use decreases (63, 64). The same relation was revealed by the studies of Arpaci et al.; furthermore they (16) suggested that there exists a significant negative correlation of mindfulness with nomophobia for both male and female subjects and mindfulness-based interventions can be effective in treating nomophobia, especially in women. Another study by the same authors (65) conclude that gender differences should be taken into account in the researches that studies mindfulness in relation with nomophobia.

However, the empirical research to support the positive influence of mindfulness in reducing nomophobia is somehow scarce and additional theoretical and research contributions to the study of the effects of mindfulness on smartphone addiction are needed (55, 66).

Recent investigations (19) showed that there is a significant positive correlation between mindfulness and psychological resilience, but a significant negative correlation between nomophobia and mindfulness. The same authors suggested that a significant mediating factor in the relationship between mindfulness and nomophobia is psychological resilience. These findings support the idea that the mindfulness-based interventions can increase psychological resilience and, consequently, help preventing nomophobia.

### 2.5 Non-pathological compulsions and nomophobia

There are not many researches that puts together the two concepts we are discussing and there is why when analyzing the relation between non-pathological compulsions and nomophobia, we can start from the observation that high levels of technology use are non-pathological unless they are linked with negative outcomes (67). The relation between nomophobia and non-pathological compulsions is very complex and it was approached differently. As some authors stated (68), in the 5th edition of DSM (69) there cannot be identified diagnostic criteria for mobile phone addiction although Internet gaming disorder is considered to be similar to gambling disorder. Starting from this, there were identified studies in this area (70) that used the diagnostic criteria for pathological gambling and/or substance dependence when analyzing nomophobia but this approach seems to be insufficiently theoretically substantiated. Other specialists in the area claim that smartphone addiction or nomophobia, is defined as a non-pathological behavioral addiction seen as extreme or problematic smartphone use (71). In this respect, it was our intention to put together the concept into the proposed model.

Additionally, in choosing this factor, non-pathological compulsions, in our study, we were inspired by work in the clinical psychology literature focusing on the association between obsessions, compulsions and nomophobia. According to this, individuals who show greater obsessive behavior (such as checking the smartphone very often) will have moderately more anxiety from “not being able to communicate with others” and/or “giving up convenience” [(9), p. 12]. Furthermore, other studies [(28), p. 7] pointed out that “sensitivity is a strong predictor of nomophobia along with obsession-compulsion, and the number of hours of smartphone use per day.” Very recent researches (72) showed that dysfunctional obsessive beliefs contribute to the occurrence of high levels of nomophobia. With all these findings in mind, involving clinical variables, we wanted to investigate the role of a non-clinical factor from the same spectrum, such as non-pathological compulsions in the occurrence of nomophobia taking into account that our study was conducted in a non-clinical setting, university environment with the participation of university students.

### 2.6 Explanatory model of nomophobia in the current study

Based on findings from previous studies and using hierarchical regression analysis, we have proposed an explanatory model of nomophobia, in which predictors were introduced into the equation according to their importance revealed in the scientific literature (21). So, a closer review of previous research has shown that the first factors considered important in explaining nomophobia were *rumination* and FoMO (*fear of missing out*). In support of this, some authors have emphasized that rumination extensively predicted excessive smartphone use (44) but there is a scarcity of research examining the mechanisms and correlations that underly this relation. Based on the work of Arpaci & Gundogan (19), we have chosen *mindfulness* as the third factor we introduced into the regression equation. The above-mentioned authors concluded that nomophobia exhibits a notable adverse correlation with mindfulness, while other authors (55, 66) have indicated that the empirical research to support the positive influence of mindfulness in reducing nomophobia is somehow scarce and further theoretical frameworks and research contributions are required to enhance our understanding of how mindfulness affects smartphone addiction. The final step of the hierarchical regression equation involved *non-pathological compulsions*. The reasons for introducing this factor at the end of the regression equation were due to the fact that there are no direct studies that examine the relationship between non-pathological compulsions and nomophobia, but rather we drew from clinical psychology and the differential diagnostic criteria of the DSM, 5th edition. From this latter perspective, evidences are provided by Gonçalves et al. (28) and García-Masip et al. (72) who revealed that the development of nomophobia involves the impact of obsession-compulsion tendencies and dysfunctional obsessive beliefs.

## 3 Methodology

The study relied on a quantitative approach for data gathering, employing a questionnaire-based survey.

### 3.1 Hypotheses of current study

Drawing from prior research, we formulated the following hypothesis:

*H1a*: Rumination, fear of missing out (FoMO), and non-pathological compulsions are positively related to nomophobia, meaning that as university students have higher levels of rumination, fear of missing out FoMO, and non-pathological compulsions, they will have higher levels of nomophobia.

*H1b*: Mindfulness is negatively related to nomophobia, meaning that for university students, increasing the level of mindfulness decreases nomophobia.

*H2*: Rumination, fear of missing (FoMO), mindfulness, and non-pathological compulsions form a significant predictive model of nomophobia.

*H3*: There is a mediation effect of fear of missing (FoMO) on the relationship between rumination and nomophobia, in that rumination affects nomophobia both directly and indirectly through fear of missing out. Consequently, a high level of rumination is linked to a higher level of nomophobia, both directly and indirectly through the intensification of fear of missing out, which in turn is associated with an increased level of nomophobia.

*H4*: There are significant gender differences in nomophobia, meaning that female participants aged 19 to 25 years have a higher fear level of being left without a phone compared to male participants.

*H5*: Nomophobia significantly varies depending on daily time spent using smartphone by study participants. In this respect, participants who utilized their phones for over 3 h daily are expected to exhibit a greater level of nomophobia.

### 3.2 Population and sampling

One hundred ninety-four university students from Iasi, Romania participated in a survey specifically designed to gather information on nomophobia, rumination, fear of missing out (FOMO), mindfulness, and non-pathological compulsions. Some demographic and relevant data were collected at the end of the questionnaire: participants’ age, gender, occupation, daily time spent on smartphones (<1 h, 1–3 h, 3–5 h, > 5 h), the most commonly used activities on smartphones (social networking, WhatsApp, Internet searching, SMS text messages, phone calls, games, music, other activities) and time since they own a smartphone (<1 year, 2 years, 3 years, 4 years, > 5 years). The questionnaire was on-line accessed using Google Forms.

This study utilized convenience sampling, implying that only students who were readily accessible and available were included in the participant group. An *a priori* power analysis was carried out using G*Power version 3.1.9.7 (73) to ascertain the minimum sample size necessary for testing the study hypothesis. The findings revealed that a sample size of *N* = 84 was required to achieve 80% power for detecting a medium effect, with a significance criterion set at α = 0.05, in the context of Correlation: Bivariate normal model. Thus, the attained sample size of *N* = 194 effectively tests the study hypothesis. Out of the participants, 43.39% were male, while 56.61% were female. The average age of the participants was21.03 (SDage = 1.78, range = 19–25). Prior to participation, all individuals provided informed consent for inclusion in the study. This research was conducted in compliance with the Declaration of Helsinki, and the protocol received approval from the Ethics Committee of the Faculty of Psychology and Educational Sciences at the “Alexandru Ioan Cuza” University of Iasi, Romania, in March 2023.

### 3.3 Instrumentation

The research design encompassed five concepts measured through dedicated questionnaires that were pretested on Romanian participants. Further sections offer detailed information regarding the reliability of these instruments. Therefore, *nomophobia* was measured using the *Nomophobia Questionnaire* (NMP-Q) developed by Yildirim & Correia in 2015. This tool covers 20 items and answers were collected on a seven-step Likert-type scale, where 1 means total disagree and 7 total agree. A high level on this scale represents greater involvement in using mobile phone, a state of heightened anxiety when access to this device is not allowed.

In order to measure *rumination,* we used the Rumination Scale, which includes 12 items from the *Rumination-Reflection Questionnaire* by Trapnell and Campbell (74). This scale describes the extent to which a person tends to think excessively about past events. Items 6, 9 and 10 of this scale are inverted items. Participants were required to indicate their level of agreement with each statement on a 5-point Likert scale ranging from 1 (strongly disagree) to 5 (strongly agree). A high score on this scale is a higher occurrence of negative thoughts, more negative interpretations of information, events and the future (75).

The scale used in our study to measure mindfulness was *The Mindful Attention Awareness Scale* (MAAS), developed by Brown and Ryan (76). This assessment focus on dispositional mindfulness, emphasizing the presence or absence of attention and awareness concerning the present moment. It specifically targets these aspects rather than attributes such as: acceptance, trust, empathy, gratitude, or other factors typically linked with mindfulness. The tool comprises 15 items, requiring participants to rate their agreement on a scale ranging from 1 to 6: 1 (almost always) to 6 (almost never) concerning each statement. A high score on this scale means increased attention to what is happening now, a high level of well-being, better life satisfaction and the presence of positive emotions, while low-score participants show symptoms such as depression, anxiety, negative emotions, anger and loneliness (76).

To assess Fear of Missing Out, we utilized the Fear of Missing Out Scale (FOMOS) developed by Przybylski (56). The tool includes 10 items and asks participants to indicate the state of truth or falsehood for each statement, according to their experience. A high score on this scale represents an altered general mood, a lower level of well-being, a lower level of life satisfaction and mixed feelings in using social networks, a decrease in sleep quality and an increase in negative emotions, stress and physical symptoms (77).

Non-pathological compulsions were measured using *Compulsiveness Inventory questionnaire* developed by Kagan and Squires (78). The instrument comprises 12 items developed to measure compulsive behavior that is normal among the population. A high score on this scale represents a larger occurrence of normal compulsive behaviors (clothes and home cleaning, checking doors/windows several times), compared to a low score on this scale which refers to lack of interest in the activities listed above (78).

### 3.4 Reliability

To assess the internal consistency of each scale utilized in the research, a pilot study was conducted, and the questionnaires were applied to 30 students. The results indicated that Cronbach’s alpha had values ranging from 0.63 to 0.94, as follows: 0.94 for Nomophobia Questionnaire (NMP-Q), 0.88 for Rumination-Reflection Questionnaire, 0.82 for The Mindful Attention Awareness Scale (MAAS), 0.81 for Fear of Missing Out Scale (FOMOS), and 0.63 for the Compulsiveness Inventory questionnaire. These findings demonstrated that the proposed instruments are reliable and that they can be used to collect data.

## 4 Data analysis procedure

The data was analyzed using the SPSS software program, Version 20.0. The study involved distributing the Google Form link to 250 students from various faculties at Iasi University, Romania. Out of the 250 completed questionnaires, only 194 responses that successfully passed the SPSS screening and cleaning procedures and were included in the current analysis.

In this investigation, hypotheses were formulated to explore potential statistically significant relationships among the variables. Consequently, methods like Pearson’s correlation, hierarchical regression, and mediation analysis employing Hayes’s PROCESS tool are deemed suitable for testing these hypotheses. According to Chan et al. (79) for samples which fall between 50 and 300, we need a z range between −3.29 and +3.29 in order to have a normal distribution. As shown in Table 1, the statistical data indicate a normal distribution.

**Table 1:** Results of the normal distribution.

Descriptive statistics									
	N	Minimum	Maximum	Mean	Std. Deviation	Skewness		Kurtosis	
	Statistic	Statistic	Statistic	Statistic	Statistic	Statistic	Std. Error	Statistic	Std. Error
Nomophobia	194	21.00	138.00	72.3351	24.32884	0.149	0.175	−0.559	0.347
Rumination	194	21.00	60.00	41.2938	8.57405	−0.133	0.175	−0.394	0.347
FoMO	194	10.00	50.00	28.5103	7.44669	0.034	0.175	0.065	0.347
Mindfulness	194	24.00	84.00	51.5722	10.86552	−0.140	0.175	0.105	0.347
Non-pathological compulsions	194	0.95	1.26	1.0913	0.07239	−0.038	0.175	−0.675	0.347
Valid N (listwise)	194								

## 5 Findings

Firstly, we hypothesized that it is possible to identify a set of relationships between rumination, fear of missing out (FoMO), mindfulness, non-pathological compulsions and nomophobia. More precisely, we suggested that rumination, fear of missing out (FoMO), and non-pathological compulsions are positively related to nomophobia, while mindfulness is negatively related to nomophobia.

As shown in Table 2, statistical data revealed the existence of a significant positive correlation between rumination and nomophobia, *r*(194) = 0.343, **p < 0.01, and a significant positive correlation between the fear of missing out and nomophobia, *r*(194) = 0.539, **p < 0.01. In other words, young people aged 19 to 25 who get high scores on rumination and fear of missing out tend to get high scores on nomophobia, too. Our findings are consistent with previous research conducted by Khoo and Yang (44), Lian et al. (54), and Cheng et al. (55) who found that there is a direct, positive and significant relationship between rumination and nomophobia. Additionally Hoşgör and Hoşgör (18), demonstrated a noteworthy moderate positive and direct relationship between nomophobia and fear of missing out. Their study revealed that fear of missing out accounted for 30% of nomophobia. Regarding the relationship between non-pathological compulsions and nomophobia, the Pearson correlation analysis indicated a significant negative correlation between non-pathological compulsions and nomophobia, *r*(194) = −0.251, **p < 0.01, which is an unexpected result, meaning that Romanian students who get high scores on non-pathological compulsions tend to get low scores on nomophobia. In this respect, we consider that the empirical research to support the positive relationship of non-pathological compulsions and nomophobia is somehow scarce and additional theoretical and research contributions are needed to study the impact of non-pathological compulsions on smartphone addiction.

**Table 2:** Correlations among variables.

Variables	1	2	3	4	5
Nomophobia	1	0.343**	0.539**	0.147*	−0.251**
Rumination		1	0.438**	0.338**	−0.108
Fear of missing out (FoMO)			1	0.328**	−0.127
Mindfulness				1	0.39
Non-pathological compulsions					1

*p < 0.05, two-tailed. **p < 0.01, two-tailed. *n* = 194.

The results concerning relationship between mindfulness and nomophobia pointed out a significant positive correlation between mindfulness and nomophobia, *r*(194) = 0.147, *p = 0.041. This means that the students participating in this research with high scores on mindfulness are more likely to have high scores on nomophobia; this result is in contrast with several earlier studies. Existing research suggests that while mindfulness increases, the effect of nomophobia on addictive smartphone use decreases (63, 64). The same relation was revealed by the studies of Arpaci et al. (65); furthermore they (65) suggested that there exists a significant negative correlation between mindfulness and nomophobia for both male and female subjects.

**Figure 1:**
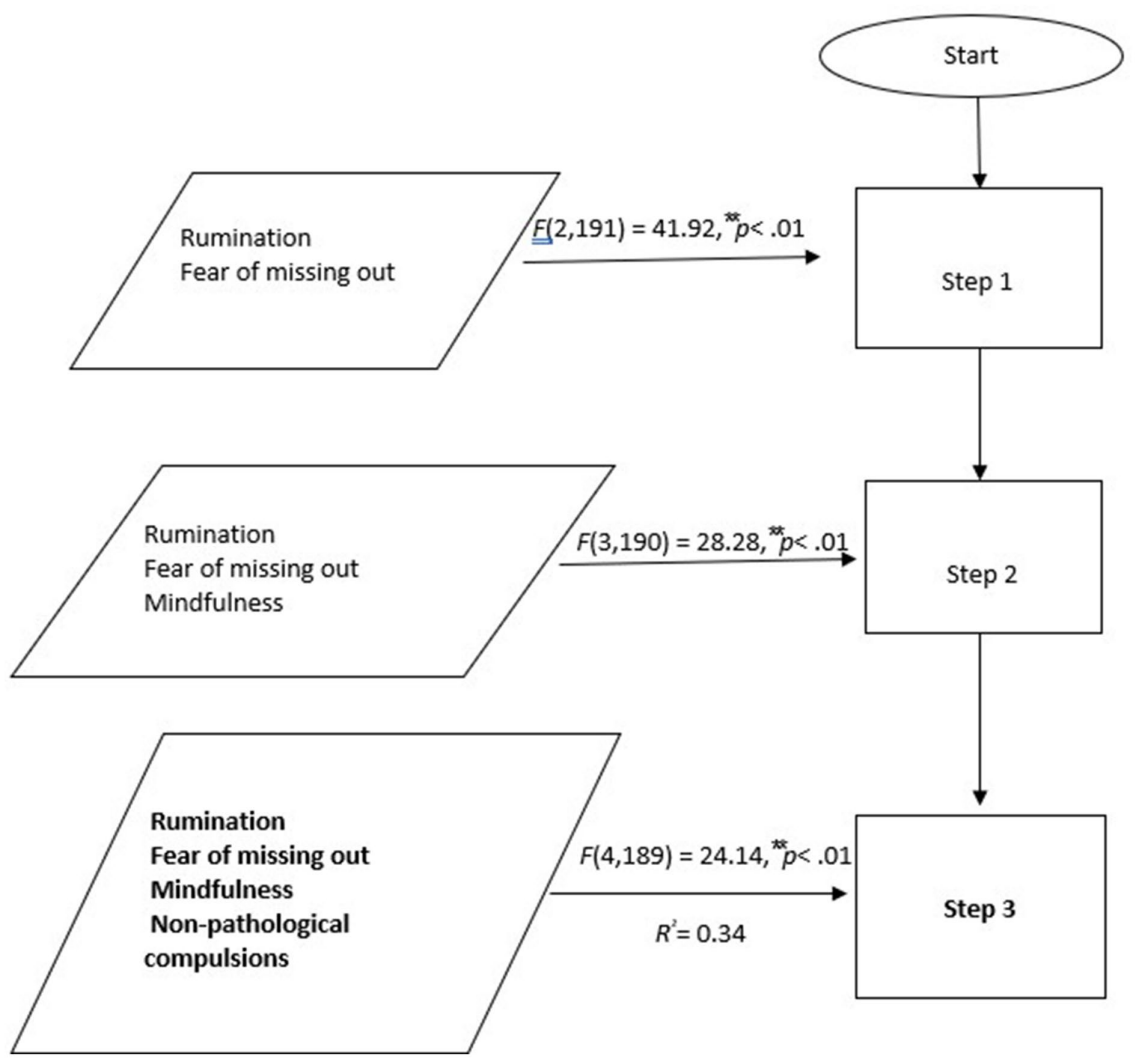
Regression diagram.

To examine the efficacy of explanatory models concerning nomophobia involving rumination, fear of missing out, mindfulness, and non-pathological compulsion, a three-stage hierarchical regression analysis was carried out (Figure 1).

Therefore, rumination and fear of missing out were entered at stage one of regression. Mindfulness was added at stage two and non-pathological compulsion at stage three. Variables were introduced in this order due to their importance revealed in previous studies. The significant values of standardized coefficients and changes in *R^2^* were examined to assess the interaction terms’ capacity to elucidate variance beyond what is already explained by the main effects in the equation. Intercorrelations between the multiple regression variables were reported in Table 2 and the regression statistics are in Table 3.

**Table 3:** Summary of hierarchical regression analysis for variables predicting nomophobia.

Variable	*β*	*t*	sr^2^	*R*	*R* ^2^	∆*R*^2^
Step 1				0.55**	0.31**	0.305**
Rumination	0.132	1.97*	0.12			
Fear of missing out	0.481	7.17**	0.43			
Step 2				0.56	0.31	0.004
Rumination	0.148	2.15*	0.13			
Fear of missing out	0.496	7.22**	0.44			
Mindfulness	−0.066	−1.00	−0.06			
Step 3				0.58*	0.34*	0.029*
Rumination	0.132	1.95*	0.12			
Fear of missing out	0.475	7.00**	0.41			
Mindfulness	−0.047	−0.72	−0.04			
Non-pathological compulsion	−0.174	−2.90*	−0.17			

*p < 0.05, two-tailed. **p < 0.01, two-tailed. *n* = 194.

The hierarchical multiple regression revealed that at stage one, rumination and fear of missing out contributed in a significant manner to the regression model, *F*(2,191) = 41.92, **p < 0.01, and described for 31% of the variation in nomophobia. On introducing mindfulness, variables explained an additional 0.4% in the variation in nomophobia and this change in *R^2^* was significant, *F*(3,190) = 28.28, **p < 0.01. Adding non-pathological compulsion to the regression equation, the model explained an additional 3% of the variation in nomophobia and this change in *R^2^* was significant, *F*(4,189) = 24.14, **p < 0.01.

The findings suggested that Model 3, comprising rumination, fear of missing out, mindfulness, and non-pathological compulsions, stands as the most accurate explanatory model. It displayed an adjusted coefficient of determination *R^2^* = 0.34, indicating that the variables within Model 3 collectively account for 34% of the variance in nomophobia. The highest explanatory contribution for nomophobia is given by fear of missing out, followed by non-pathological compulsions and rumination. Additionally, the effect size indicators for significant predictors were: *r_sp_* = 0.41 for fear of missing out, *r_sp_* = −0.17 for non-pathological compulsions, and *r_sp_* = 0.12 for rumination. Therefore, the effects are medium (fear of missing out) and weak (non-pathological compulsions and rumination). Nomophobia is equal to 82 + 1.55*fear of missing out +0.37*rumination – 58.53*non-pathological compulsions, where fear of missing out is coded in dichotomous units 1 = true and 2 = false, non-pathological compulsions is also coded in dichotomous units 1 = true and 2 = false, and rumination is coded in units ranging from 5 = agree to 1 = disagree. These values indicate that as fear of missing out increases by one unit, nomophobia increases by 1.55 units. Also, as rumination increases by one unit, nomophobia increases by 0.37 units. Finally, as non-pathological compulsion increases by one unit, nomophobia decreases 58.53 units.

**Figure 2:**
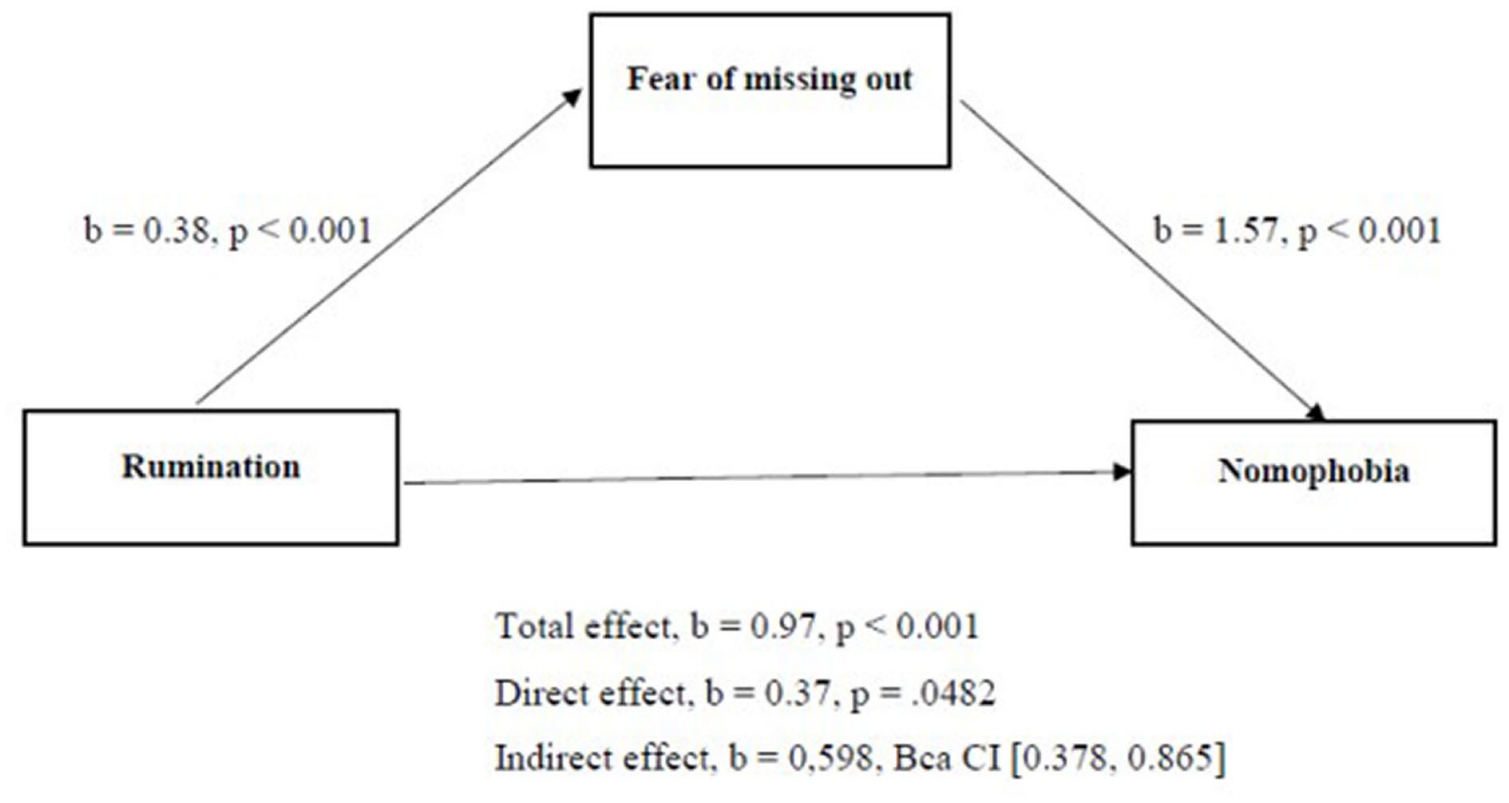
Model of rumination as predictor of nomophobia, mediated by fear of missing out.

The nomophobia explanatory model that we have examined so far has unveiled a direct relationship between rumination, fear of missing out, mindfulness, and non-pathological compulsions (the predictors) and nomophobia (the outcome). Taking into consideration the complexity of nomophobia and starting from previous evidence (17, 18, 44, 54, 55), it’s obvious that the relationships among the factors explored in the study are multifaceted. Consequently, we hypothesized that the relationship between rumination and nomophobia is not only a direct effect, but it operates through an increase in fear of missing out (FoMO). The hypothesis was examined through a mediation analysis using Hayes’s PROCESS tool (Figure 2).

According to study findings, there was highlighted a significant indirect effect of rumination on nomophobia through fear of missing out, *b* = 0.598, Bca CI [0.378, 0.865], and similarly *K^2^* = 0.206, 95% Bca CI [0.132, 0.285]. *K^2^* is bounded between 0 and 1, so we can interpret this as the indirect effect being about 20.6% of the maximum value that it could have been, which is a medium and meaningful mediation effect. A high level of rumination leads to a high level of nomophobia, both directly and indirectly through increasing the fear of missing out, which leads to a higher level of nomophobia. Sobel mediation test values (*z* = 4.89, **p < 0.01) reconfirmed the importance of the fear of missing out factor as a mediator of the relationship between rumination and nomophobia. As we can see in Figure 2, the statistical analysis has demonstrated the presence of partial mediation within the proposed conceptual model. Specifically, it indicates that fear of missing out acts as a partial mediator in the relationship between rumination and nomophobia. It’s encouraging to note that these relationships are in line with the predicted direction.

As gender has been taken into account in previous works (65) in relation to some intervention models in treating nomophobia, showing their effectiveness especially in women, the study examined the difference in the intensity of nomophobia for man and woman participants (Table 4). We hypothesized that nomophobia is more present in young females compared to males. The outcomes from the Independent-Samples T Test indicate significant differences in nomophobia based on gender, *t*(192) = −4.555, **p < 0.01. Young female aged 19 to 25 years obtained higher scores on nomophobia (MD = 79.38) compared to young males of the same age (MD = 64.18).

**Table 4:** Gender differences in nomophobia.

Variable *n*	M SD	*t*	df *p*
Nomophobia	- 4.555	192	**p < 0.01
Male	90	64.18	24.92
Female	104	79.38	21.53

*p < 0.05, two-tailed. **p < 0.01, two-tailed.

A final refining of the analysis of nomophobia involved the time spent by students on mobile phone. Statistical data (Table 5) indicated that there is a significant difference between participants who use mobile phone more than 3 h a day and those who use it less than 3 h a day regarding the level of nomophobia, *t*(192) = −4.276, **p < 0.01. As the time of using mobile phone increases to more than 3 h a day, the level of nomophobia also increases (MD = 78.52), compared to the situation when time spent on the mobile phone is less than 3 h a day (MD = 64.06).

**Table 5:** Time spent on mobile phones and nomophobia.

Variable *n*	M SD	t df	*p*
Nomophobia	- 4.276	192	**p < 0.01
Less than 3 h/day	83	64.06	22.33
More than 3 h/day	111	78.52	24.00

*p < 0.05, two-tailed. **p < 0.01, two-tailed.

## 6 Discussion

Nowadays, in the empire of modern technologies, it has become really challenging to decipher the codes of human behavior. We can easily notice a variety of contrasting effects in using mobile phones, computers, the Internet and all the applications. All these are supposed to make our lives easier, but on the contrary, sometimes we experience undesirable effects on us, such as restlessness, fear and worry. In other words, there are times when, for example, using the phone leads to negative cognitive and affective reactions in terms of personal characteristics and achievements, social integration, effective interaction with the world around us and the possibility of being in continuous development. Such a negative affective reaction is *nomophobia.* In the scientific literature, several studies (15, 22, 27–30, 32, 80) have examined nomophobia as a factor underlying behavioral changes or negative affective reactions, and far fewer have explored what actually determines people to develop such a phobia. In light of these facts, our study wanted to highlight some triggers of nomophobia and to clarify the nature of the relationships between those predictors and nomophobia.

Our current research aimed to elucidate specific psychological factors that contribute to the presence of nomophobia among students within the Romanian university context. On this multidimensional phenomenon of nomophobia, fear of missing out (FoMO), non-pathological compulsions, and rumination, included in a regression model, in this order, played the most important role, together explaining 34% of nomophobia variance. Furthermore, the fear of missing out (FoMO) has the greatest explanatory power of nomophobia. In terms of the interpretation of these connections, there existed a direct, positive, and significant correlation between fear of missing out, rumination, and nomophobia, and a direct, negative, and significant association for non-pathological compulsions and nomophobia. This means that as fear of missing out (FoMO) and rumination increase, nomophobia also increases, and as non-pathological compulsions increase, nomophobia decreases. In other words, among Romanian students, the greater the uncertainty related to fear of missing out (FoMO) a call, a message (6) or social network updates, the greater fear of being out of mobile phone access will be. Complementary, the more students show a tendency to think passively and repetitively about personal current feeling states, own characteristics and the possible causes and consequences of these symptoms (45), the higher the level of nomophobia will be. These results confirm the hypotheses formulated in this study, and are also consistent with previous literature.

In their study, Gezgin et al. (80) identified a moderately positive relationship between nomophobia and levels of fear of missing out (FoMO). As well, Hoşgör and Hoşgör (18) showed that there is a moderately significant association and a positive direct relationship between fear of missing out and nomophobia, and that fear of missing out accounts for 30% of nomophobia. Indeed, in a study involving Chinese college students (81), highlighted that smartphone use for entertainment purposes and concerns about missing out on enjoyable experiences with friends were linked to susceptibility to nomophobia. Concerning the relationship between rumination and nomophobia, it involves a double sense. On one side, a closer review of previous studies indicates that rumination extensively predicts excessive smartphone use (44), but on the other side, in modern societies, there’s a shift in understanding rumination–it’s not only seen through a cognitive lens but also through the lens of human-device interaction. This expanded view, acknowledges how our contemplations intersect with and are influenced by our interactions with technology (82).

.Looking to map out the concepts and relationships underlying nomophobia, we have recognized that our explanatory model, focusing on the direct impact of predictors on the dependent variable, only addresses one facet of the issue. Based on previous studies (17, 18, 44, 54, 55, 83), we tested a mediator model in which the relationship between rumination and nomophobia is mediated by fear of missing out. So, we found a significant indirect effect of rumination on nomophobia through fear of missing out, which means that a high level of rumination predicts a high level of nomophobia, both directly and indirectly through increasing the fear of missing out, which, in turn, predicts a higher level of nomophobia.

Given the extent to which nomophobia has taken hold in the last few years, a comparative analysis of the variability of this phenomenon along relevant structural dimensions can improve our understanding of the concept. In the current study we have confirmed a prevalence of nomophobia in young females aged 19–25 years in comparison with young males of the same age. These findings are in line with the hypotheses formulated and are also supported by previous studies, some authors suggesting that nomophobia is present in both men and women, but specific intervention models in treating nomophobia may be more effective especially in women (16, 65).

The current study’s results align with Gonçalves, Dias, and Correia’s findings (28), indicating that the number of hours spent using smartphones daily serves as a strong predictor of nomophobia. Specifically, our study suggests that young individuals who use mobile phones for more than 3 h per day tend to exhibit higher levels of nomophobia. Basically, we are dealing here with a matter of duration in using smartphones which has been associated with terms such as *excessive use* or *overuse* of smartphones and smartphone addiction (60).

Finally, we can state that, based on the current study findings, the ideas, implicitly, the research hypotheses have been confirmed. Thus, we can emphasize the existence of a predictive model of nomophobia, in which the factors fear of missing out (FoMO), non-pathological compulsions, and rumination, included in a regression model, in this order, played the most important role. Additionally, this research has demonstrated the significant mediating effect of factor fear of missing out (FoMO) on the relationship between rumination and nomophobia. Given that we did not identify studies that put together the above-mentioned psychological factors into explanatory model of nomophobia, we believe that present results help to a better understanding of nomophobia and could guide a potential intervention to dilute this type of phobia.

## 7 Limitations of the study

The initial premise was rooted in previous studies indicating the prevalence of nomophobia, primarily among adolescents and university students. However, the significant limitation of the current study stemmed from the use of a study group confined to one university’s subjects. However, this does not diminish the value of the data obtained on the selected sample of participants. The results found in the present study may express a pattern that can certainly be replicated in other groups of university students. Indeed, the methodology’s reliance on a Google Form questionnaire might have impacted the objectivity and potentially introduced desirability biases in the participants’ responses to the questions. The adaptation of the questionnaires to the cultural context could be an extra limitation of the study, despite the fact that the scales showed good psychometric properties. Nevertheless, the outcomes of this study have provided significant insights and have led to comprehensive conclusions regarding potential explanations for nomophobia among young individuals.

## 8 Recommendations for future research

This study paves the way for future research endeavors and offers opportunities for further interpretation and exploration concerning nomophobia, for example, detailing the impact of mindfulness on nomophobia using of a multidimensional scale covering the facets of the concept of mindfulness. Furthermore, it is essential that in a future methodological investigation on nomophobia, the role of other predictor/mediator variables should also be taken into account. It would be interesting to test and clarify the relationships between depression, anxiety, psychological resilience, boredom, panic disorder, nervousness, welfare satisfaction with life and nomophobia. Ultimately, further research efforts will be essential to explore and understand nomophobia more comprehensively, using a longitudinal design with multiple measurements to more accurately capture the dynamics of this phenomenon.

## 9 Conclusion

According to the current research findings, fear of being without a mobile phone or losing mobile phone contact especially found in young people is predicted at 34% by rumination, fear of missing out, mindfulness, and non-pathological compulsion. Among these factors, the highest explanatory contribution for nomophobia is given by fear of missing out, which has a direct impact on the phenomenon, but also mediates the relationship between rumination and nomophobia. In other words, on one hand, a high level of rumination leads to a high level of nomophobia, both directly and indirectly through increasing the fear of missing out, which, on the other hand, leads to a higher level of nomophobia. In addition, the study indicates the prevalence of nomophobia in women, and draws attention to time duration in using smartphones daily.

## Data availability statement

The raw data supporting the conclusions of this article will be made available by the authors, without undue reservation.

## Author contributions

CC, AM, and RA contributed to conception and design of the study and wrote sections of the manuscript. AM organized the database and performed the statistical analysis. CC wrote the first draft of the manuscript. All authors contributed to manuscript revision, read, and approved the submitted version.
